# Scientific production of Brazilian dermatology: analysis of abstracts submitted at the Annual Meeting of the American Academy of Dermatology (2005 to 2013) and those eventually published*

**DOI:** 10.1590/abd1806-4841.20165042

**Published:** 2016

**Authors:** Nicole França Holmo, Cinthia Rosane Orasmo, Silvio Alencar Marques

**Affiliations:** 1 Private clinic – São Paulo (SP), Brasil; 2 Private clinic– Bauru (SP), Brasil; 3 Universidade Estadual Paulista "Júlio de Mesquita Filho" (Unesp) – Botucatu (SP), Brasil]

**Keywords:** Scientific publication indicators, Congresses, Dermatology

## Abstract

In the last decade the presence of Brazilian physicians in International Meetings of Dermatology has been expressive. In parallel it has also been expressive the submission of poster abstracts in those Meetings. Considering the meetings from 2005 to 2013, 379 posters were presented in meetings of the American Academy of Dermatology. Brazilian universities were the origin of 59.9%. The Brazilian Society of Dermatology's recognized residency programs were the origin of 69.9% of the presented posters. Considering the period from 2005 to 2010 (n = 165 posters) the papers effectively published were 19 (11.5%).

The participation of Brazilian physicians in international congresses of Dermatology is growing in recent years. At the same time, the number of posters submitted and accepted for presentation at these congresses is also increasing. It is known that most of the posters presented at congresses in Brazil do not result in effective publications in indexed journals, as well as the scientific production of Brazilian Dermatology is scarcely studied, quantitatively and qualitatively.^1-3^ The authors aimed at quantifying and classifying the Brazilian submissions accepted for presentation at the Annual Meeting of the American Academy of Dermatology from 2005 to 2013, as well as to identify how many of these posters have resulted in complete articles effectively published in journals indexed in national and international databases. A descriptive-analytical, cross-sectional and retrospective study of all abstracts (poster abstracts) presented and published in the supplements of the Journal of the American Academy of Dermatology (JAAD) from 2005 to 2013 concerning the meetings of the respective years was conducted. Studies that included at least one Brazilian author, with a national link, were considered for analysis, regardless they belong or not to university institutions. The abstracts were classified according to the following criteria: institution of origin (IO), if University Service or not, if residency program recognized by the Brazilian Society of Dermatology (SBD) or not, topic of interest according to the classification of the JAAD supplement, type of investigation or if case report. We searched the papers presented between 2005 and 2010 published in journals indexed in LILACS, SciELO and MEDLINE-PubMed databases. The published studies were classified according to: IO, if originated in a residency program recognized by the SBD, publication in international or national journals and if the paper underwent modifications in its title or authorship.^4^

Three hundred seventy-nine posters were presented, increasing from only 15 in 2005 to 86 in 2013. According to the IO: 59.9% was presented by a university institution, and most of them was conducted by the Federal University of Rio de Janeiro (UFRJ) = 7.8% of 379; followed by the University of São Paulo (USP) = 3.9%; and the Fluminense Federal University (UFF) = 3.4%. Presentations by non-university services/ clinics totaled 40.1%, performed by the General Polyclinic of Rio de Janeiro = 11.1% of 379; by the Institute of Dermatology Professor Rubem D. Azulay = 10.3%; and by the Otávio Macedo Clinic = 4.5%. The papers originated in residency programs recognized by SBD constituted 69.9% of the total, and those from non-accredited services accounted for 30.1%. Regarding the topic of interest of the study, 13.2% of 379 was interpreted as related to non-infectious clinical dermatology; focusing on bacterial/parasitic infection, 11.1%; focusing on fungal infection, 5.3%; and related to laser therapy, 5.0% of 379 studies. Regarding the type of papers presented: case reports/case series accounted for 55.4% of 379 studies; therapeutic study, 19.5%; and cross-sectional research study, 12.4% of the total. As to the publication, 19 of the 165 papers presented in the period between 2005 and 2010, equivalent to 11.5% of the total, were identified as effectively published. In international journals, there were 16/19 (84.2%) publications, among which 9 (47.3%) came from therapeutic studies. Among the published works, 9/19 (47.4%) were conducted in university institutions. The authorship, at the time of publication, was modified by additions/substitutions, in relation to what appeared in the original abstract, in 94.7% of the posters. The titles of the posters, at the time of publication, were partially modified in 31.5% of the studies without, however, changing the matrix of the original title contained in the abstract. The journals that most received such publications were Dermatologic Surgery (15.8%, i.e., 3/19); Brazilian Annals of Dermatology, also with 15.8% of publications; International Journal of Dermatology, with 10.5% (2/19); and Journal of the European Academy of Dermatology and Venereology, also with 10.5% of the publications, followed by several journals with a single published study each.

**Graph 1:**
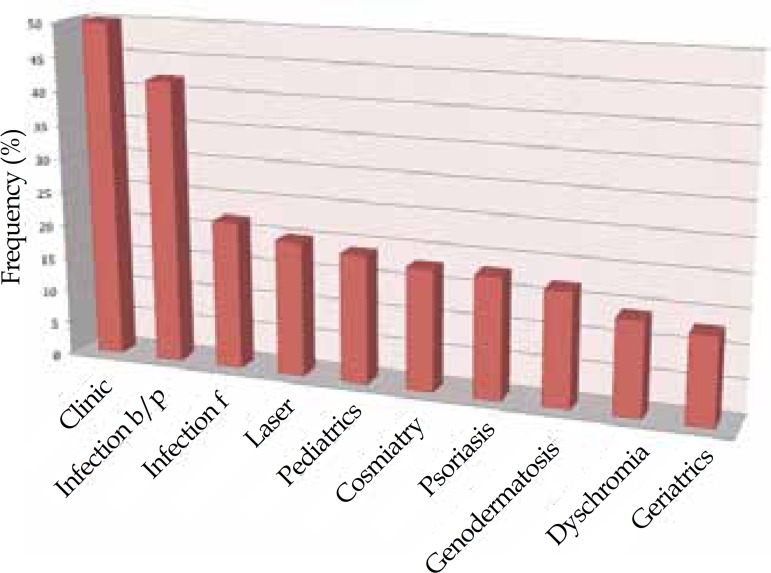
Posters presented at the Annual Meeting of the American Academy of Dermatology according to the topic of interest (period from 2005 to 2013)

**Graph 2:**
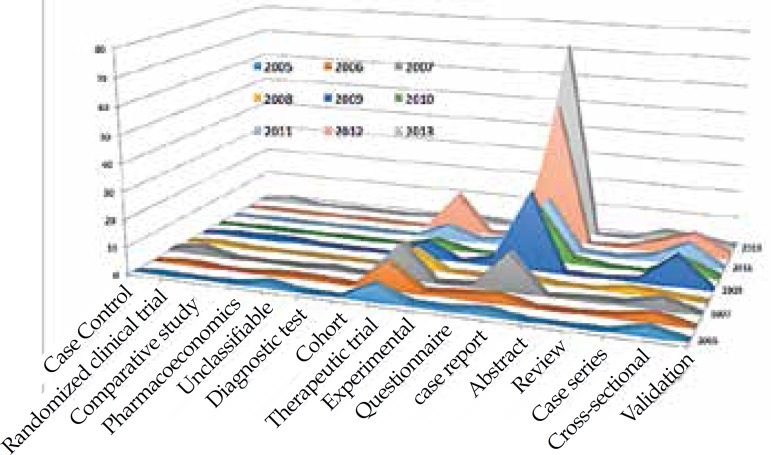
Posters presented at the Annual Meeting of the American Academy of Dermatology according to the type of study conducted (period from 2005 to 2013)

In conclusion, the authors consider as expressive the number of studies accepted and effectively presented (n = 379) in the Meetings of the American Academy in the period assessed, and this number increased over the years. Attention is drawn to the percentage of non-university services (40.1%) and, mainly, those from services/clinics that were not residency programs recognized by SBD, which accounted for 30.1% of the papers presented. This latter percentage may express a possible growth in the performance and production of services and clinics outside SBD and may also be the result of the American Academy's policy of not blocking submissions from services non-accredited by officially representative Societies of Dermatology in their respective countries.

The topics of interest were consistent with the tradition of Brazilian Dermatology, focusing on Clinical Dermatology and infectious diseases, and even in the case of an international congress, the case reports predominated. The percentage (11.5%) of studies that consolidated as a publication, from 2005 to 2010, can be considered as intermediary compared with the only two national studies that address this theme: 6.32% of the posters presented at the National Congress Of Angiology and Vascular Surgery and 26.6% of the posters presented at the National Congress of Orthopedics. ^5,6^

The authors draw attention to numerous papers of great interest, judging by the title and abstracts contained in the supplements consulted, which, for unknown reasons, are no longer published.

As a limitation of the present study, it should be noted that it only analyzes the submissions to the Annual Meetings of the American Academy of Dermatology. The extension of the study to other international congresses of Dermatology would give greater breadth and solidity to the objectives and conclusions presented here.

## References

[1] Gerbase AC, Ponzio HA, Bernardi CDV, Bassanessi SL, Stumpf MK (1990). Produção científica do 44º Congresso
Brasileiro de Dermatologia. An Bras Dermatol.

[2] Marques SA, Miot HA, Abbade LPF (2008). Produção científica publicada nos Anais
Brasileiros de Dermatologia (2003-2007). An Bras Dermatol.

[3] Martins MCA, Carneiro MGLN, Utzing JB, Neta ELK, Pachnicki MA, Castro CCS (2012). Produção científica de dermatologistas
brasileiros nos últimos 25 anos, nos cinco jornais de maior impacto
na Dermatologia. An Bras Dermatol.

[4] Belinchón I, Ramos JM (2008). Scientific output of Spanish dermatology departments in
international journals, 1997-2006. Actas Dermosifiliogr.

[5] Yoshida WB, Holmo NF, Corregliano GT, Baldon KM, Silva NS (2008). Indexed publications generated from abstracts of angiology and
vascular surgery congresses in Brazil. J Vasc Bras.

[6] Ejnisman L, Gomes GS, de Oliveira RG, Malavolta EA, Gobbi RG, de Camargo OP (2013). Publication rates of papers presented at the Brazilian Orthopedic
Meeting. Acta Ortop Bras.

